# Interspecies Somatic Cell Nuclear Transfer Is Dependent on Compatible Mitochondrial DNA and Reprogramming Factors

**DOI:** 10.1371/journal.pone.0014805

**Published:** 2011-04-27

**Authors:** Yan Jiang, Richard Kelly, Amy Peters, Helena Fulka, Adam Dickinson, Daniel A. Mitchell, Justin C. St. John

**Affiliations:** 1 Mitochondrial and Reproductive Genetics Group, Clinical Sciences Research Institute, Warwick Medical School, Coventry, United Kingdom; 2 Centre for Reproduction and Development, Monash Institute of Medical Research, Monash University, Victoria, Australia; 3 Mitochondrial and Reproductive Genetics Group, The Medical School, The University of Birmingham, Edgbaston, Birmingham, United Kingdom; 4 Department of Biology of Reproduction, Institute of Animal Science, Prague, Czech Republic; Brunel University, United Kingdom

## Abstract

Interspecies somatic cell nuclear transfer (iSCNT) involves the transfer of a nucleus or cell from one species into the cytoplasm of an enucleated oocyte from another. Once activated, reconstructed oocytes can be cultured *in vitro* to blastocyst, the final stage of preimplantation development. However, they often arrest during the early stages of preimplantation development; fail to reprogramme the somatic nucleus; and eliminate the accompanying donor cell's mitochondrial DNA (mtDNA) in favour of the recipient oocyte's genetically more divergent population. This last point has consequences for the production of ATP by the electron transfer chain, which is encoded by nuclear and mtDNA. Using a murine-porcine interspecies model, we investigated the importance of nuclear-cytoplasmic compatibility on successful development. Initially, we transferred murine fetal fibroblasts into enucleated porcine oocytes, which resulted in extremely low blastocyst rates (0.48%); and failure to replicate nuclear DNA and express Oct-4, the key marker of reprogramming. Using allele specific-PCR, we detected peak levels of murine mtDNA at 0.14±0.055% of total mtDNA at the 2-cell embryo stage and then at ever-decreasing levels to the blastocyst stage (<0.001%). Furthermore, these embryos had an overall mtDNA profile similar to porcine embryos. We then depleted porcine oocytes of their mtDNA using 10 µM 2′,3′-dideoxycytidine and transferred murine somatic cells along with murine embryonic stem cell extract, which expressed key pluripotent genes associated with reprogramming and contained mitochondria, into these oocytes. Blastocyst rates increased significantly (3.38%) compared to embryos generated from non-supplemented oocytes (P<0.01). They also had significantly more murine mtDNA at the 2-cell stage than the non-supplemented embryos, which was maintained throughout early preimplantation development. At later stages, these embryos possessed 49.99±2.97% murine mtDNA. They also exhibited an mtDNA profile similar to murine preimplantation embryos. Overall, these data demonstrate that the addition of species compatible mtDNA and reprogramming factors improves developmental outcomes for iSCNT embryos.

## Introduction

Somatic cell nuclear transfer (SCNT), which involves the transfer of an adult or fetal cell into an enucleated oocyte, utilises the cytoplasmic factors already present within the oocyte to reprogramme the somatic cell [1]. Following incubation of the somatic cell in the recipient oocyte and subsequent activation, the resultant embryos can be cultured to the blastocyst stage, the final stage of preimplantation development. At this stage, cells can be isolated from the inner cell mass (ICM) and cultured in vitro as potential ‘personalised’ embryonic stem cells (ESCs; [2]). The expanding colonies of pluripotent ESCs then have the potential to develop into any cell type of the body. Such approaches have led to the generation of murine models of haematopoiesis [3], regenerative strategies for Parkinson′s disease [4] and non-human primate ESC lines [5].

The use of SCNT to generate human ESC lines modelling disease is, however, restricted by ethical considerations and access to human oocytes for research purposes [6]. Consequently, animal oocytes have been proposed as the most suitable alternative to host human somatic nuclei, i.e. interspecies/admixed SCNT (iSCNT; [7]). Indeed, studies using iSCNT have reported development to the blastocyst stage following the transfer of human [8], sheep, porcine and monkey nuclei into bovine oocytes [9] and macaque nuclei into rabbit oocytes [10]. There is also a single report of the generation of several human ESC lines following the transfer of human nuclei into rabbit oocytes [11]. However, a number of reports have highlighted, amongst other factors, the failure of many iSCNT embryos to initiate and progress further than embryonic genome activation (EGA) [12] most likely through unsuccessful reprogramming and initiation of embryonic transcription [13].

In the vast majority of cases, SCNT also results in the mixing of chromosomal (somatic cell) and mitochondrial DNA (mtDNA; somatic cell and recipient oocyte) from different sources. MtDNA is located within the inner membrane of the mitochondrion and is present in nearly all eukaryotic cells. It encodes 13 of the 90+subunits [14] of the electron transfer chain (ETC), which is the cell's major generator of ATP through oxidative phosphorylation (OXPHOS). In order to ensure that mature tissues and cells produce ATP at maximum efficiency, the mammalian embryo strictly regulates the transmission of mtDNA from the population present in the oocyte just prior to fertilisation [15], as is the case for those offspring generated from oocytes fertilised with sperm from the same breed or strain (intraspecific crosses). Usually each of these copies is identical (homoplasmic) as they originate from the 200 copies present in each primordial germ cell laid down just after gastrulation [16] and are then clonally expanded. Interestingly though, the process that eliminates sperm mtDNA in intraspecific crosses does not mediate its loss in interspecific crosses [17].

In SCNT embryos, the mtDNA accompanying the somatic cell is either eliminated during preimplantation development, resulting in homoplasmic transmission of recipient oocyte mtDNA, or persists resulting in heteroplasmy, a combination of donor cell and recipient oocyte mtDNA. Transmission of donor cell mtDNA ranges from 0 to 63% in preimplantation embryos [18] and 0 to 59% in live offspring [19]. This tends to be independent of whether intra- or inter-specific SCNT is performed [20]. For example, donor cell mtDNA has been detected in bovine embryos derived by both intra- [21] and inter-specific NT [18], though not in all cases [18], [19], and in caprine embryos [22] and porcine offspring [23] derived by interspecific SCNT. However, as there are sequence variations in the mtDNA coding genes for breeds within the same species, this can result in different combinations of amino acid synthesis and the degree of heteroplasmy could considerably reduce the ability of any resultant stem cells to generate sufficient ATP through OXPHOS [24].

Following iSCNT, donor cell mtDNA has been detected at the 16-cell stage in human-bovine embryos [8], the blastocyst stage in macaque-rabbit embryos [10] and in a small minority of caprine-ovine embryos (4/40; range = 2 to 20+cell embryos; [12]). However, the tendency is for donor cell mtDNA in more genetically diverse fusions to be eliminated during development, perhaps reflecting the difference in size of the mitochondrial genome between species. In porcine cells, it is approximately 16.7 kb [25] whilst the human and murine mtDNA genomes are 16.6 kb [14] and 16.2 kb [26], respectively. Furthermore, the increased genetic distance between the donor cell and the recipient oocyte could also affect nucleo-mitochondrial compatibility. To this extent, interspecies cybrid studies, where somatic cell karyoplasts were fused to enucleated cytoplasts, demonstrated that increased genetic distance between the two fusion partners resulted in decreased ATP output [27] most likely due to the nuclear-encoded polypeptides of the ETC failing to interact with the mtDNA-encoded subunits. Furthermore, nucleo-mitochondrial incompatibility could impact on mtDNA replication, which is mediated through nuclear-encoded factors. These include the mtDNA-specific DNA polymerase, Polymerase Gamma (POLG), its catalytic (POLGA) and accessory (POLGB) subunits; mitochondrial transcription factor A (TFAM) which generates the primer for replication; and Twinkle, the mtDNA-specific helicase [28].

In order to determine whether viable iSCNT blastocysts can be developed for potential stem cell derivation, we have transferred murine somatic cells into enucleated porcine oocytes. However, the porcine cytoplasm exerted considerable influence on embryo development including the failure to initiate chromosomal DNA replication and promoted the preservation of porcine rather than murine mtDNA. Depletion of porcine oocyte mtDNA and supplementation with murine ESC extract containing mitochondria and factors to promote cellular reprogramming, improved embryo development to blastocyst and karyokinesis and allowed preferential replication of murine mtDNA.

## Results

**Figure 1:**
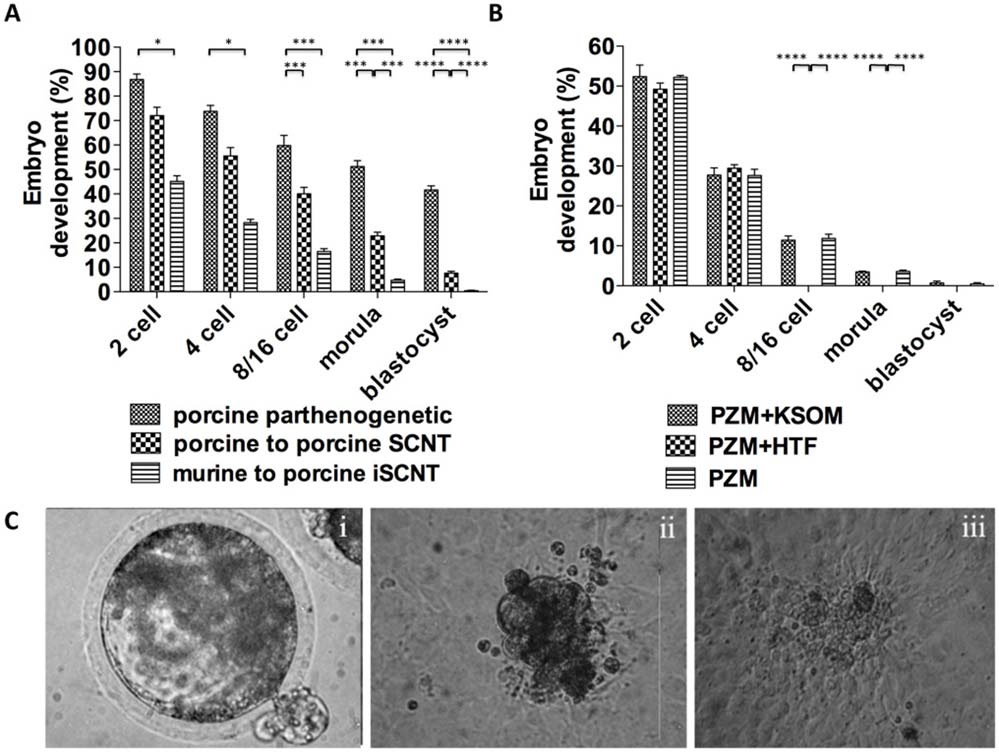
Outcomes from iSCNT. (A) Preimplantation development analysis of murine-porcine and porcine-porcine cloned embryos and porcine parthenogenetic embryos. (B) The effects of different culture systems on the iSCNT embryos in vitro development (PZM - n = 226; PZM+HTF - n = 147; PZM+KSOM - n = 132). (C) In vitro culture of iSCNT embryos: intact embryo with some protrusion (i); proliferating cells from an assisted hatched embryo after 5 days (ii) and 14 days (iii) plated on MEF feeder layers. Data are represented as mean±SEM, * indicates P<0.05, and *** indicates P<0.001 and **** indicates P<0.0001.

In order to determine the efficiency of the cytoplasm of an oocyte from one species to reprogramme a somatic nucleus from another, we performed SCNT by injecting mouse embryonic fibroblasts (MEFs) into enucleated porcine oocytes. In total, we performed 2080 iSCNT manipulations using three different embryo culture systems: porcine zygote medium (PZM); PZM+Human tubal fluid (HTF); and PZM+Potassium simplex optimized medium (KSOM). Overall, 10 (mean±SEM = 0.48±0.253%) of the interspecies reconstructions developed to blastocyst (see Figure 1A). This contrasts with a blastocyst development rate of 9.4% following the intra-specific transfer of porcine cumulus cells into enucleated porcine oocytes. Both these outcomes were significantly lower than for parthenogenetically activated porcine oocytes (P<0.0001), of which 41.1% developed to blastocyst. Subdividing the reconstructed oocytes into 3 cohorts allowed us to determine whether, when the embryonic genome had been activated, media specific to the donor cell species would enhance development. Reconstructed embryos were transferred from PZM into HTF (n = 147) or KSMO (n = 132) at the 2/4-cell stage; or were maintained in PZM (n = 226). Although there was no development to blastocyst for those embryos cultured in HTF, similar blastocyst rates were recorded for those cultured in KSOM (0.79%; n = 2) and PZM-only (0.463%; n = 2) (Figure 1B). As a result, PZM was used for all subsequent experiments.

In order to determine whether iSCNT blastocysts (n = 5) showed conventional morphological characteristics, we examined their ability to expand in vitro and whether the murine embryonic genome could mediate the thinning of the zona pellucida and the hatching process. Blastocysts on day 7.5 post-reconstruction demonstrated limited thinning of the zona pellucida and only partial embryo protrusion and were unable to fully complete hatching without manual assistance (Figure 1Ci). We plated whole blastocysts (n = 3) stripped of their zona pellucida onto MEF feeder layers in order to promote the expansion of cell number. We observed that two of these blastocysts gave rise to clumps of cells that were clearly visible after 3 to 5 days (Figure 1Cii) but only one expanded for a further 9 days (Figure 1Ciii) before ceasing to expand further and disintegrating.

**Figure 2:**
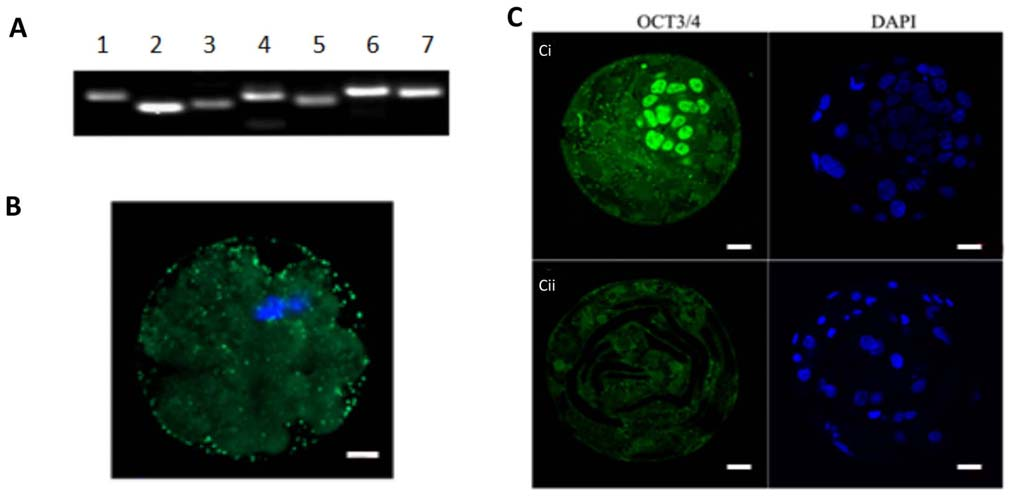
Chromosomal DNA and cytoplasmic regulation of iSCNT embryos. (A) Analysis of microsatellite markers in a single iSCNT morula after whole genome amplification. Lane 1, D8Mit242; Lane2, D19Mit10; Lane 3, D16Mit146; Lane 4, D1Mit206; Lane 5, DXMit170; Lane 6, D12Mit270; Lane 7, D4Mit178. (B) DAPI (blue) and NPC (green) staining in a murine-porcine cloned embryo undergoing cytokinesis. (C) Distribution of OCT-4 in a murine in vivo blastocyst (i); and a murine-porcine cloned blastocyst (ii). OCT-4 and DAPI are shown in green and blue, respectively. Scale bars = 20 µm.

In order to determine the genetic integrity of these embryos, we performed microsatellite DNA analysis on 7 murine chromosomes following whole genome amplification. Of 14 iSCNT morulae analysed, murine chromosomal DNA was present in only 2 of these embryos (Figure 2A). Furthermore, for those blastocysts that had been plated onto feeder layers and subsequently developed into clumps (n = 2), there was also no chromosomal DNA present (data not shown). However, in this instance, we were unable to confirm whether the DNA had not been replicated from the outset or had degraded following plating. Nevertheless, we were able to confirm that mouse chromosomal DNA had been transferred but failed to replicate, as indicated by the presence of DAPI staining in just one of the blastomeres of these multi-cellular embryos (Figure 2B). This DNA was not within a defined nuclear structure as demonstrated by the lack of nuclear pore complex (NPC) staining (Figure 2B). Consequently, many of the embryos had undergone cytokinesis but not karyokinesis. For those murine-porcine iSCNT embryos that had undergone karyokinesis, they possessed poorly arranged NPCs (Figure S1Aii), when compared with their intraspecific counterparts (Figure S1Ai), and consequently the chromatin was not part of a functional cell nucleus.

Using a monoclonal antibody specific to phosphorylated-H2A.X (p-H2A.X), which is a well-established early marker of DNA double strand breaks, it is evident that p-H2A.X was more intensely distributed in a higher proportion of blastomeres in the murine-porcine embryos (Figure S1Bii). However, *in vivo* murine (Figure S1Bi) and porcine-porcine SCNT (Figure S1Biii) embryos revealed the reduced likelihood of DNA strand breaks. In order to determine whether a porcine oocyte could reprogramme a mouse nucleus, we stained the iSCNT embryos with OCT4 and observed almost no staining (Figure 2Cii). In contrast, murine in vivo fertilised blastocysts had clearly defined staining for OCT4 in the ICM (Figure 2Ci).

**Figure 3:**
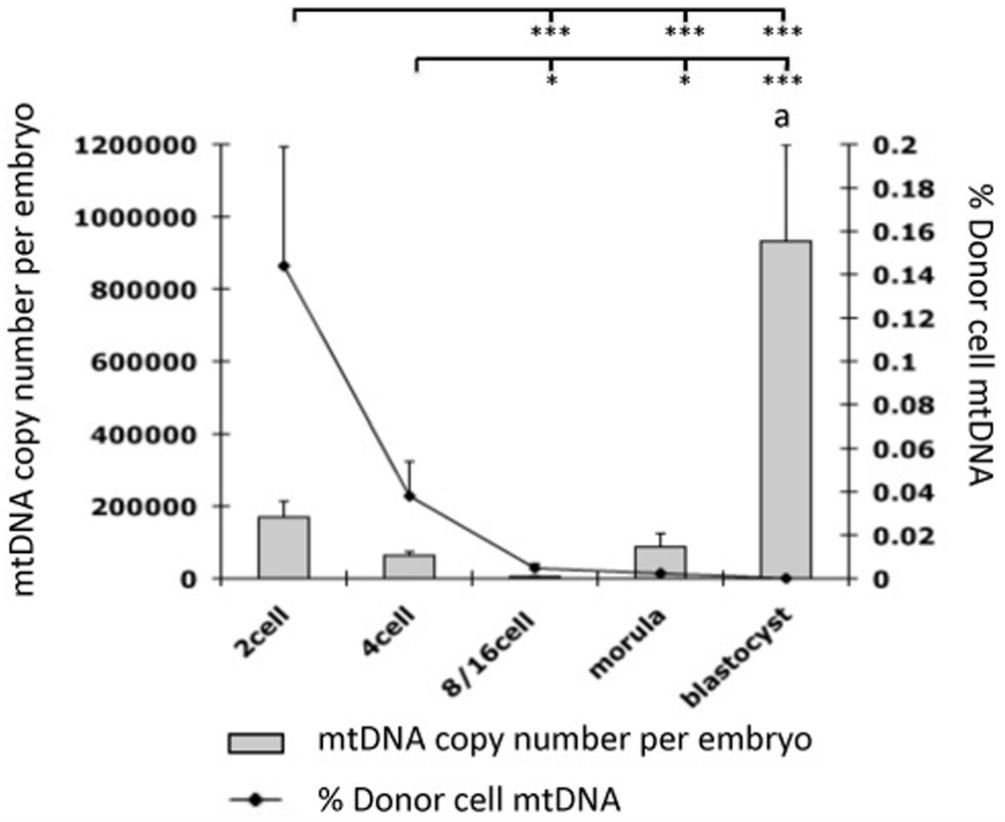
Analysis of total mtDNA copy number and donor cell contribution throughout preimplantation development of murine-porcine embryos. Superscript ^a^ indicates a significant difference of P<0.001 with all other stages of development. Mean (±SEM) donor cell mtDNA content, as assessed by AS-PCR, is expressed as a percentage of total donor cell mtDNA. P<0.05 is denoted by *; P<0.01 by **; P<0.001 by ***.

In order to determine how iSCNT embryos would regulate their mtDNA content, we assessed these embryos for their overall mtDNA content and the persistence of donor cell mtDNA (Figure 3). Using AS-PCR, it was evident that the levels of murine mtDNA decreased throughout preimplantation development up to the blastocyst stage but at ever-decreasing levels (P<0.001). The peak level was at 0.14±0.055% (mean±SEM) in 2-cell embryos, representing 243 copies of mtDNA whilst the lowest level was in blastocysts (P<0.001; Figure 3). There was an increase in the percentage of donor cell mtDNA between reconstruction and the 2-cell stage, which was not significant (p>0.05), but represented approximately a 5-fold increase in murine mtDNA over the donor MEF contribution (50±4.3 copies of mtDNA per cell). The presence of donor cell mtDNA throughout preimplantation development also confirmed that the donor cell had been transferred into the enucleated recipient oocyte. Amplification of total mtDNA by quantitative real time PCR showed a decrease in mtDNA copy number at the 4-cell stage. This is similar to that reported for porcine, but not murine, in vitro-fertilised (IVF)-embryos, followed by the initiation of mtDNA replication at the morula stage with significant increases by the blastocyst stage (P<0.001; Figure 3), again similar to porcine IVF-derived preimplantation embryos.

**Figure 4:**
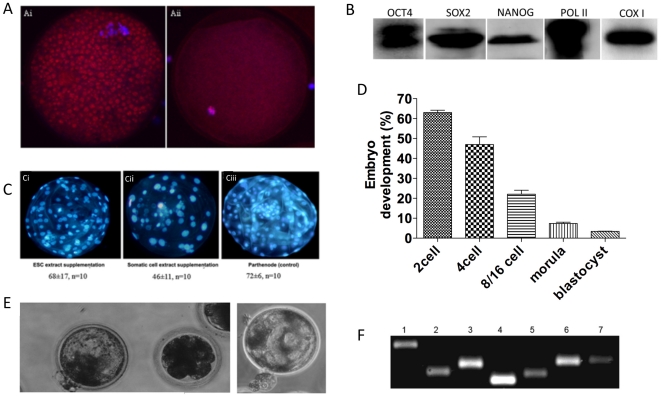
Depletion of porcine oocyte mtDNA and supplementation with murine ESC extract. (A) Analysis of mitochondrial distribution after ddC treatment for 24 hr (i) and 42 hr (ii) during IVM. Mitochondria and nuclei are stained with MitoTracker Red (red) and DAPI (blue), respectively. (B) Western blot analysis of ESC extract for protein expression of OCT3/4, SOX2, NANOG, RNA Polymerase II (POLII) and the mtDNA-encoded COXI. (C) Parthenogenetic blastocysts generated with (i) ESC extract; (ii) MEFs; or (iii) control. (D) In vitro development rates of iSCNT embryos following injection of MEF donor somatic cell and mESC extract into enucleated porcine oocytes. (E) Two expanding iSCNT blastocysts demonstrate thinning of the zona pellucida. (F) Analysis of donor cell specific microsatellite markers in an iSCNT morula. Lane 1, D8Mit242; Lane 2, D1Mit206; Lane 3, D19Mit10; Lane 4, D16Mit146; Lane 5, DXMit170; Lane 6, D4Mit178; Lane 7, D12Mit270.

We then determined whether the cytoplasm of porcine oocytes could be remodelled to host compatible populations of chromosomal and mtDNA by introducing murine cytoplasmic factors and mitochondria that would enhance development of iSCNT embryos to blastocyst. In preliminary experiments, we have demonstrated that 2′,3′-dideoxycytidine (ddC) depletes porcine oocytes of 95% of their original mtDNA content during 42 hours of IVM, which results in 20160±4625 copies of mtDNA persisting [29]. By staining oocytes during IVM with MitoTracker, a mitochondrial-specific label, we have further substantiated the depletion process by demonstrating that the vast majority of the oocyte′s mitochondria were not functional through the loss of their mtDNA genome after 42 hours (cf. Figures 4Ai and ii). Through staining with DAPI, we were also able to confirm that the oocytes were able to complete maturation and progress to Metaphase II as evidenced by the extrusion of the 1^st^ polar body (data not shown).

We then tested whether such oocytes could support preimplantation development when supplemented with either murine ESC or somatic cell extract. We screened the ESC and somatic cell extracts by microarray and determined that key genes of pluripotency were expressed or upregulated in the ESC extract. Those genes that were significantly upregulated, i.e. had>4-fold increase in expression when compared to MEF extract, included transcription factors mediating stemness; signaling molecules required for pluripotency and self-renewal; cytokines and growth factors; and other embryonic stem cell-specific genes (see Table S1). We also confirmed that OCT4, NANOG and SOX2 proteins were present in the ESC extract (Figure 4B). Furthermore, we also determined that the RNA Polymerase II (POLII), which would potentially aid reprogramming and embryonic transcription; and the mtDNA-encoded COXI were present (Figure 4B). Non-enucleated porcine oocytes were then injected with either somatic cell or ESC extract to the point that the cytoplasm was slightly distorted. The amount of ESC extract injected was equivalent to the cytoplasmic volume obtained from approximately 6350 ESCs. As well as providing a higher yield of blastocysts, the ESC extract resulted in higher numbers of blastomeres in the parthenogenetically activated oocytes and better cell organisation (Figure 4Ci), more similar to the control parthenotes (Figure 4Ciii) than following supplementation with somatic cell extract (Figure 4Cii).

**Figure 5:**
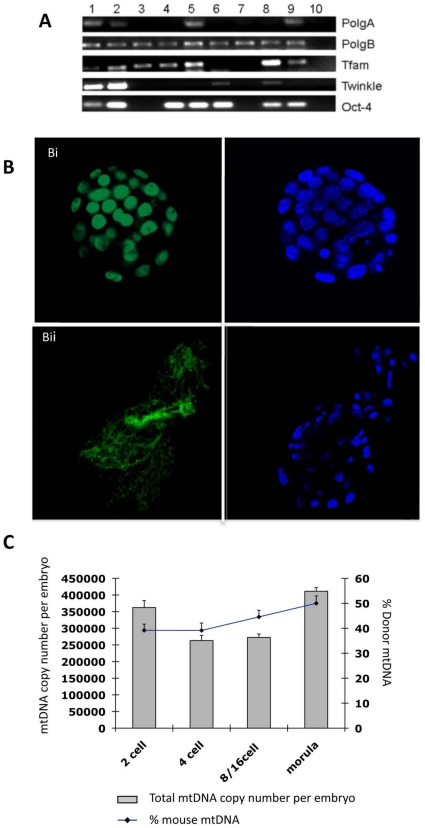
Analysis of pluripotent and mtDNA replication factors and mtDNA content in mtDNA-depleted, ESC supplemented-iSCNT embryos. (A) RT-PCR analysis for gene expression of PolgA, PolgB, Tfam, Twinkle and Oct4 in single cloned embryos and ESCs. Lane 1–10 ESCs; Lane 2–20 ESCs; Lane 3–2-cell embryo; Lane 4–4-cell embryo; Lane 5–4-cell embryo; Lane 6–8-cell embryo; Lane 7–8-cell embryo; Lane 8-morula; Lane 9-blastocyst; Lane 10-Negative control. (B) Distribution of Oct-4 in an *in vivo* fertilised-derived blastocyst (i) and a murine-porcine cloned blastocyst (ii). Oct-4 and DAPI are shown in green and blue, respectively. (C) Total mtDNA copy number and percentage of donor cell mtDNA contribution in iSCNT embryos supplemented with murine ESC extract containing mitochondria.

By injecting enucleated mtDNA-depleted porcine oocytes simultaneously with murine ESC extract and a murine somatic donor cell, we generated 207 reconstructions that produced 2-cell, morula and blastocyst stage embryos at rates of 63.288±1.16% (n = 131), 7.24±0.64% (n = 15) and 3.38±0.1% (n = 7), respectively (see Figure 4D). Although these blastocysts were able to expand in culture and induce thinning of the zona pellucida, they were not able to hatch but demonstrated slight protrusion of the zona pellucida (see Figure 4E). These developmental rates were significantly higher than somatic cell supplemented (P<0.05; data not shown) and non-supplemented iSCNT embryos (P<0.01; see Figure 1A) whilst somatic cell supplemented and non-supplemented iSCNT embryos had similar development rates (P>0.05). Through microsatellite analysis, we were able to demonstrate that they possessed murine chromosomal DNA (Figure 4F). RT-PCR analysis of a cohort of these embryos demonstrated expression of *Oct 4* (see Figure 5A) although we found no evidence of it being expressed as a protein in iSCNT embryos (Figure 5Bii) but it was present in *in vivo* fertilised-derived embryos (Figure 5Bi). AS-PCR analysis demonstrated that, during preimplantation development, donor cell mtDNA content was higher at later stages, with morula-stage embryos possessing 49.99%±2.97% (Figure 5C). The decline in mtDNA copy number during early preimplantation development was far less marked than for the non-mitochondrial supplemented embryos, which is similar to in vivo murine preimplantation embryo development. This was matched by the expression of the mtDNA-specific replication factors, which as a whole, were expressed at the 2-cell and blastocyst stages (Figure 5A).

## Discussion

The first stages of embryonic development are dependent on cytoplasmic factors that are carried over from the fertilized oocyte. These mediate a multitude of intracellular events including karyokinesis and cytokinesis, which are essential for complete cell division [30]. Once the embryonic genome is activated, intracellular events are synthesised from the newly formed embryonic genome [12], [13]. True intergeneric hybrids, which are derived from a full set of sperm and oocyte haploid chromosomes from two distinct species, often fail to develop past the onset of EGA [31]. However, there are notable exceptions such as the crossing of female horses and male donkeys that give rise to mules [32]. Even though iSCNT does not normally result in an abnormal chromosomal complement, which is common to some true hybrid combinations, the somatic cell will still be dependent on the recipient oocyte's cytoplasmic factors until the embryonic genome is activated as following fertilization [33]–[35]. Indeed, our data highlight that the donor somatic nucleus requires species-specific cytoplasmic factors to ensure chromosomal DNA is faithfully replicated so that, following cell division, each newly formed blastomere possesses intact chromosomal DNA. We also show that NPC organisation is chaotic and these cells have increased incidence of DNA strand breaks. This dysfunction of the NPC may have influenced the nucleo-cytoplasmic exchange of factors that are essential for embryonic progression and may explain the aberrant developmental phenotypes we have observed. In addition, as these embryos developed, the vast majority arrested whilst a few generated blastocyst-like structures that possessed little or no DNA. Expanded blastocysts were able to initiate embryo hatching although the signal to complete the process was absent most likely due to chromatin fragmentation and aberrant reprogramming of the murine genome. Once plated, these embryos formed clumps that could survive in ESC culture for only a very limited period of time.

Reprogramming of the somatic cell is also dependent on factors present within the oocyte's cytoplasm [1]. However, in our genetically diverse model, there is little reprogramming of the somatic cell during preimplantation development, as determined by the lack of OCT-4 protein expression (Figure 2C ii). Porcine double nuclear transfer, i.e. the transfer of a somatic cell into an enucleated oocyte, its activation and subsequent transfer of the pronuclei into an enucleated zygote, has demonstrated that reprogramming is not always dependent on a single incubation in an oocyte's cytoplasm [36]. In our model, where the porcine cytoplasmic factors are incompatible with murine donor DNA, this is simply not achievable in one round of iSCNT. This failure to reprogramme is similar to recent outcomes demonstrated in human-bovine and human-rabbit iSCNT [13]. However, the introduction of ESC extract containing factors associated with reprogramming, namely OCT-4, SOX 2 and NANOG, along with POL II, which mediates RNA transcription, resulted in an increase in development to blastocyst and blastomeres possessing DNA. Some of these preimplantation embryos expressed *Oct-4* mRNA suggesting that a degree of reprogramming had taken place, although little discernible protein was observed.

The dominant function of the recipient oocyte's cytoplasm is further demonstrated by the mtDNA content in the embryos generated from non-supplemented oocytes being consistent with patterns observed in porcine IVF embryos. In porcine IVF embryos, a significant reduction in mtDNA copy number takes place from the 4-cell stage [29] with similar events having been reported in bovine oocytes [37]. The loss of mtDNA copy number from the 4-cell to 16-cell stage in porcine IVF embryos is in spite of the upregulation in the expression of the mitochondrial DNA-specific polymerase, *PolgA*, at the 4-cell stage only. This increase in expression for *PolgA* is in line with embryonic genome activation and is most likely a transcriptional turnover event [29]. In the mouse, the embryonic genome is activated at the 2-cell stage and is associated with a burst of mtDNA replication but not an actual increase in mtDNA copy number [38], although we observe a 5-fold increase in murine mtDNA copy number in our murine-porcine 2-cell embryos based on the amount of mtDNA accompanying the donor cell at reconstruction. However, the mtDNA profile for porcine [29] and bovine [37] embryos is very different to murine [39] embryos, where murine mtDNA copy number remains consistent throughout preimplantation development [39] and our embryos reflect the porcine profile.

The dominance of the recipient oocyte's cytoplasm is further highlighted by the loss of murine mtDNA as the non-supplemented embryos progressed to the blastocyst stage. In intra- and inter-specific SCNT preimplantation embryos, where donor cell mtDNA has been reduced to residual levels through mtDNA-specific depletion (<0.02% of the original mtDNA content), the preferential replication of donor cell mtDNA is attributed to the persistent expression of mtDNA-specific replication factors, such as POLGA, POLGB and TFAM [12]. This contrasts with in vitro fertilised embryos, which first express these factors at the blastocyst stage [12], [29], [37]. However, in more genetically diverse models, such as caprine-ovine iSCNT embryos, both POLGA and TFAM were also upregulated [12] but, as with the non-supplemented murine-porcine crosses, donor cell mtDNA was almost totally eliminated by the later stages of preimplantation development. This suggests that the donor cell was replicating the genetically more diverse recipient oocyte mtDNA throughout preimplantation development, as also evident in our more divergent model at the blastocyst stage.

Nevertheless, our strategy of depleting recipient oocytes to approximately 5% of their original mtDNA content and supplementing them with murine ESC extract containing mitochondria not only led to increased development to blastocyst, but vastly increased the amount of murine mtDNA present throughout preimplantation development. We chose to use mitochondria from undifferentiated ESCs, as they are similar to those of the oocyte and preimplantation embryo [reviewed in 40]. They are spherical and oval in shape with poorly developed cristae indicating an inability to support OXPHOS whilst differentiated cells possess elongated mitochondria with well-developed cristae capable of supporting OXPHOS. Consequently, as shown in Figure 4C, murine ESC extract containing immature mitochondria enhanced embryonic development in porcine oocytes and their development to blastocyst whilst murine somatic cell extract containing more mature mitochondria had an adverse effect on preimplantation development. This outcome is supported by other reports, which, for example have demonstrated that development rates to blastocyst of murine parthenogenetically activated oocytes supplemented with murine somatic mitochondria were lower than in non-supplemented controls and those supplemented with oocyte cytoplasm [41].

In the iSCNT-supplemented embryos, there were slight decreases in overall mtDNA copy number between the 2 and 4 cell stages of preimplantation development. However, murine mtDNA levels remained constant during this period with levels then increasing throughout the remaining stages of preimplantation development. This is consistent with events in intra- and interspecific SCNT embryos, where the persistence and/or increases in donor cell mtDNA copy number is mediated by the upregulation of the mtDNA-specific replication factors [12], [42]. Nevertheless, in our model, there is active replication for both murine and porcine mtDNA resulting in near equal contributions from both sources at the final stages of preimplantation development. However, the more constant levels of mtDNA throughout preimplantation development with a distinctive increase at later stages are similar to those outcomes associated with murine in vivo embryos [39] suggesting that the addition of species-specific cytoplasmic factors along with donor cell compatible mtDNA mediates cytoplasmic regulation in a manner more similar to the donor cell's genetic background rather than the recipient oocyte's.

The use of mtDNA depletion and supplementation protocols to improve embryonic development is supported by studies analysing the effect of the genetic distance between the donor cell and the recipient oocyte. To this extent, the mixing of mtDNA genomes from different breeds within a species induces mild transitions in sequence variation between the coding genes. This is reflected in interspecific bovine [43] and porcine [24] SCNT offspring where the sequence variants between the donor cell and recipient oocyte mtDNA populations result in different amino acids being generated within one offspring. These offspring seem to survive parturition though in many cases such combinations fail to survive the first six months post-natally [44]. The degree of conformational change in the subunits of the ETC induced by two divergent mtDNA genomes will determine their suitability for ATP production, and is most likely a function of the genetic distance between the 2 sources of mtDNA. A small-scale study comparing the effects of haplotype between two breeds of cattle demonstrated that one mtDNA lineage produced a greater number of blastocysts following SCNT [45] whilst the other showed improved developmental competence following IVF [46]. Experiments using hand-made cloning, where the somatic donor cell can be fused to one or more enucleated oocytes, clarifies matters further by demonstrating that the donor cell favours a slightly genetically more diverse mtDNA haplotype to that of its own [47]. This is characteristic of development to the blastocyst stage and full term, which would require cells to be fully functional and to generate appropriate levels of ATP [47]. In ovine SCNT-embryos, a divergence up to 0.04% between the two mtDNA genomes is tolerable for development to blastocyst. However, increasing genetic distance 10-fold by transferring caprine somatic cells into ovine oocytes resulted in a few embryos progressing past embryonic genome activation with no development to blastocyst being achieved [12].

The effects of diverse mtDNA- and nuclear-encoded genes of the ETC will be most apparent once interspecies embryos have completed EGA and they attempt to assemble functional ETCs. As the embryo develops towards blastocyst, it becomes increasingly dependent on ATP generated through OXPHOS rather than glycolysis [48] and, in more genetically diverse fusions, will thus not produce sufficient ATP. A similar outcome is demonstrated by interspecies somatic cell cybrids, where, for example, the fusion between a murine karyoplast and rat cytoplast results in efficient replication, transcription and translation of rat mtDNA by murine nuclear-encoded factors but OXPHOS function is compromised [49]. Similar outcomes have been observed in cybrids from human and other closely related primates [50]. Furthermore, any resultant ESC model of disease harbouring a mutation related to a specific disorder, that might be derived in this manner, will also have OXPHOS deficiency resulting in other functional inefficiencies. This would introduce multiple experimental variables into the model that would allow false conclusions to be drawn [51].

These studies demonstrate that iSCNT is restricted by genetic distance and that successful development to blastocyst for the derivation of ESCs is likely to be highly dependent on compatible cytoplasmic factors. Amongst others, these will be essential to mediate DNA replication and reprogramming. The ability of the reconstructed oocyte to regulate mtDNA content in a manner similar to the donor cell's preferred oocyte background demonstrates coordinated nucleo-mitochondrial interactions and would allow functional ETCs to be assembled for the generation of ATP through OXPHOS. This will be essential for blastocyst formation and for the subsequent derivation of ESCs if such an approach is to be used.

## Materials and Methods

All chemicals and reagents were purchased from Sigma Chemical Company (Poole, Dorset, UK) unless otherwise stated.

### Collection of ovaries and oocyte *in vitro* maturation (IVM)

Ovaries were collected from a local slaughterhouse and transported to the laboratory in a saline solution at 37°C. Within 4 hr, antral follicles with diameters of 3 to 6mm were aspirated and cumulus oocyte complex (COCs) with evenly granulated cytoplasm and surrounded by more than 3 layers of cumulus cells were selected for IVM as described before [29] with some modifications. Approximately 50 COCs were put into each well of a 4-well dish (Nunc, Roskilde, Denmark) containing TCM-199 medium (Gibco, Bristol, UK) supplemented with PVA (0.1%), D-glucose (3.05 mM), sodium pyruvate (0.91 mM), penicillin (75 µg/ml), streptomycin (50 µg/ml), cysteine (0.57 mM), LH (0.5 µg/ml), FSH (0.5 µg/ml), epidermal growth factor (10 µg/ml) with and without 10 µM 2′,3′-dideoxycytidine (ddC) and were cultured for 42 hr at 39°C, 5% CO_2_ in air, as described in [29].

### Preparation of donor cells

Murine embryonic fibroblasts (MEFs, C57BL/6J) and murine ESCs (CCE/R) were used as donor cells for the reconstruction of inter-specific nuclear transfer embryos. Porcine cumulus cells were used as control donor cells to generate intra-specific cloned embryos. MEFs were cultured in high glucose Dulbecco modified Eagle′s medium (DMEM) with 10% foetal bovine serum (FBS), 2 mM L-glutamine (Invitrogen Life Technologies, Paisley, UK), 1% non-essential amino acids (NEAA; Invitrogen Life Technologies) and 1% penicillin/streptomycin solution. CCE/R mESCs were cultured in high glucose DMEM with 15% ESC screened FBS (Hyclone, Cramlington, UK), 1% penicillin/streptomycin solution, 2mM L-glutamine, 1% NEAA, 0.1 mM β-mercaptoethanol and 1000 U/ml leukaemia inhibitory factor (LIF; Chemicon, Hampshire, UK).

### Preparation of ESC extracts

To prepare ESC extracts, cells were washed twice in PBS supplemented with 0.1% protease inhibitor cocktail (Promega, Southampton, UK), sedimented at 1200 rpm then sonicated on ice using a sonicator fitted with a 3mm diameter probe until all cell and nuclei were lysed. The lysate was cleared at 13000 rpm for 15 min at 4°C, which ensures that mitochondria are intact [52]. Supernatant was frozen in beads with 2% glycerol and stored in liquid nitrogen prior to use for injection. One bead was used for protein quantification using the BCA assay (Pierce, Thermo Scientific, Paisley, UK).

### Preparation of the enucleated oocytes for nuclear transfer

At 42 hr post onset of maturation, oocytes were exposed to HEPES buffered M199 media containing 300 IU/ml hyaluronidase and then pipetted for 2 min to remove cumulus cells. Denuded oocytes, which possessed normal appearing metaphase plates and the first polar body were chosen for enucleation. The oocytes were incubated in manipulation medium containing HEPES buffered M199 supplemented with 5 µg/ml cytochalasin B for 5 min before enucleation at 39°C. The metaphase plate and the first polar body were visualized using an inverted microscope equipped with a polarized light condenser (Oosight, CRI, Cornwall, UK). Under the high contrast spindle viewer system, the metaphase spindle was enucleated using a pipette (inner diameter, 15 µm) connected to a Piezo-Electric-Microinjector (PEM, Prime Tech, Ibaraki, Japan). A minimum amount of cytoplasm was removed and complete enucleation was confirmed by observing the extracted spindle. A pipette with an inner diameter of 10 µm was used to inject a single donor cell (±ESC extract) directly into the porcine cytoplasm using PEM.

### Reconstructed oocyte activation and embryo culture

Reconstructed oocytes were activated 2 hr after injection, using the calcium ionophore A23187 (5 µM) for 5 min followed by incubation in the presence of 6-dimethylaminopurine (6-DMAP, 2.5 mM) for 3.5 hr. The embryos were then cultured in PZM, as described in [53] for the first 2 days at 39°C, 5% CO_2_ in air and then in KSOM (Millipore, Buckinghamshire, UK) or in Human Tubal fluid (HTF; Millipore, Buckinghamshire, UK) for the following 4 days at 37°C, 5% CO_2_ in air; or for the whole duration in PZM at 39°C, 5% CO_2_ in air. The intra-specific NT embryos were cultured in PZM for 7 days at 39°C, 5% CO_2_ in air.

### Mitochondrial staining

Oocytes and embryos were placed in PZM medium containing 500 µM MitoTracker Red (Invitrogen Life Technologies) and incubated in the dark for 30 min. After staining, the oocytes and embryos were washed in fresh medium at least 3 times then cultured in fresh medium for another 30 min to remove excess dye.

### Immunocytochemistry

Embryos or cells were fixed in 3.7% formaldehyde for 15 min, permeabilized with 0.5% (v/v) Triton X-100, and blocked with 3% BSA in phosphate-buffered saline (PBS). Subsequently, embryos were incubated with primary antibody as follows: 1 µg/mL anti-Oct3/4 (Santa Cruz Biotechnology, Santa Cruz, CA, USA) or 0.5 µg/mL anti-nuclear pore complex (Covance, Berkeley, CA, USA) or 0.5 µg/mL anti-H2A.X (Millipore) at 4°C overnight. After several rinses with 0.1% TritonX-100, samples were labelled with the appropriate secondary antibody Alexa Fluor 488 anti-mouse for 1 hr, counterstained with DAPI (Vector Labs, Peterborough, UK), and viewed by confocal/laser scanning microscopy (LSM, Carl Zeiss, Jena, Germany). Each channel was adjusted to remove the effects of bleed before the images were taken. For comparative analysis of protein expression in embryos or cells through confocal microscopy, the auto gain function was switched off and the same gain and photomutiplier settings were used for each sample scanned and analyzed.

### Western blotting

ESC lysates were reduced prior to SDS-PAGE by the addition of 5% β-mercaptoethanol to 15 µg total protein and boiled for 5 min at 95°C. Protein was run on a 10% polyacrylamide gel for 1 hr at 110 V then transferred onto a Hybond nitrocellulose membrane for 1 hr at 125 mA. Subsequently, membranes were blocked with 5% (w/v) milk powder in 0.05% Tween-20 buffer and slowly agitated for 1 hr at room temperature. After washing extensively in Tris-buffered saline (TBS) for 40 min, the blot was incubated in primary antibody at 4°C overnight. The antibodies used were 0.4 µg/mL anti-Nanog (Abcam, Cambridge, UK), 1 µg/mL anti-RNA Polymerase II (POLII; Abcam), 0.3 µg/mL anti-SOX2 (Abcam) and 1 µg/mL anti-Oct3/4 (Santa Cruz) and anti-COXI (Invitrogen). The blots were again washed in TBS for 40 min prior to secondary antibody incubation with either, rabbit anti-mouse IgG, and goat anti-rabbit IgG conjugated to horseradish peroxidease (0.2 µg/mL) for 1 hr. Finally, the membranes were washed for 10 min three times. Protein bands were subsequently developed on an Agfa CURIX**-**60 automatic film developer for visualization (Agfa-Gevaert, Brentford, UK).

### Sample collection and real time PCR analysis for mtDNA copy number

Nuclear transfer embryos were collected from various developmental stages. Each of the embryos was isolated and placed in 20 µl of distilled water and stored at -80°C. The embryos were thawed at 95°C for 5 min to inactivate Proteinase K. For the total mtDNA copy number, absolute quantitative real time PCR was performed using SensiMix (Bioline, London, UK) in a Rotergene-3000 real time PCR machine (Corbett Research, Cambridge, UK). Primers TF and TR (Table 1) were designed at mitochondrial Cytochrome B (CytB) homologous sites for both murine (accession no: NC005089) and porcine (accession no: NC000845) mtDNA. Conventional PCR was performed to obtain purified PCR product, which was used to produce standards consisting of 10-fold serial dilutions. For real time PCR, initial denaturation was performed at 95°C for 15 min, followed by 50 cycles of: denaturation at 95°C for 10 sec; annealing at 55°C for 15 sec; and extension at 72°C for 15 sec. Data were acquired in the FAM/SYBR channel during the extension phase. The second acquisition phase of 78°C was programmed to allow measurements of fluorescence from specific product only.

**Table 1:** Primers used for Real time and AS-PCR.

Primer PairCombinations	Sequences (5′–3′)	Annealing Temperature	Productsize
TFTR	TACTAGGAGACCCAGACAAC AGATGGAGGCTAGTTGGCC	55°C	314 bp
CO1FCO3R	GAGGACAAATATCCATTCTGAGG GGCCAACTAGCCTCCATCT	56°C	665 bp
CO2FCO3R	AGGAGACCCAGACAACTACA GGCCAACTAGCCTCCATCT	56°C	319 bp
AS-FAMTR	GAAGGTGACCAAGTTCATGCTCTAGT AGCCAACCTACTTATCT AGATGGAGGCTAGTTGGCC	56°C	109 bp
D1Mit206FD1Mit206R	TGAGGCACCTTTGTATTCAGC CCAGATGTCTTTGAACATTCTCC	55°C	125 bp
D4Mit178FD4Mit178R	GCCCTGAAGGTAAATCAGTAACT GCTCAGGAGGTACATTGCCT	57°C	146 bp
D8Mit242FD8Mit242R	TGTGCAACCAATTTCTTCCA CCCATGATTTATTCAGACTGAGG	57°C	165 bp
D12Mit270FD12Mit270R	AGGCATCTTTTTGAATAGTTTTATAC ATTAAGGCATTGGTAAAGTGATATA	53°C	146 bp
D16Mit146FD16Mit146R	AGAGAGAGAGTATGTGTCTTCCAGA GCAGATCCCTAAGAAATCAGAAG	57°C	120 bp
D19Mit10FD19Mit10R	GCCTTTAAGCCAGTCAAGACA CCAGTCTGGACTTGTGAATGA	55°C	149 bp
DXMit170FDXMit170R	TGCAGGCACTAACAGTGAGG TAGTTTCACTGTGCCATTGTATACA	57°C	114 bp
OCT-4FOCT-4R	AAGAGAAAGCGAACTAGCATT GGCAGAGGAAAGGATACAGC	55°C	235 bp
TFAMFTFAMR	GCATACAAAGAAGCTGTGAG GTTATATGCTGAACGAGGTC	53°C	165 bp
POLGAFPOLGAR	GGACCTCCCTTAGAGAGGGA AGCATGCCAGCCAGAGTCACT	60°C	188 bp
POLGBFPOLGBR	ACAGTGCCTTCAGGTTAGTC ACTCCAATCTGAGCAAGACC	55°C	214 bp
TwinkleFTwinkleR	AACGAAGCAAAGGGAAGGAT TCTGGAGCTTCAGCAAACCT	54°C	350 bp

### Allele specific PCR

To determine the ratio of donor mitochondrial DNA in preimplantation embryos, allele specific PCR was performed using 0.7 of embryo DNA (14 µl out of 20 µl volume per embryo). This was amplified in 50 µl reactions containing 1X PCR buffer, 1.5 µM MgCl_2_, 200 µM dNTPs and 2U BioTaq (Bioline), 0.5 µM of each forward (CO1F) and reverse (CO3R) primer (Table 1) to produce 665 bp of the mtDNA-encoded Cyt B gene. Reactions were run in an MJ Research PTC-200 thermocycler (GRI, Braintree, UK). Conditions were 95°C for 5 min, followed by 35 cycles of 94°C for 45 sec, 56°C for 30 sec and 72°C for 70 sec, with a final extension phase at 72°C for 5 min. In order to produce a higher quantity of product from a single embryo for AS-PCR analysis, a new forward primer (CO2F) was designed within the sequence of the CytB PCR amplicon to allow further PCR amplification of both the murine and the porcine CytB gene. For donor cell specific mtDNA detection, a forward primer (AS-FAM) was designed to incorporate the SNPs at the 3′ end, which were complimentary to a region of the murine CytB gene only, and a FAM fluorphore tag sequence was added to the 5′ end (Table 1). The reverse primer was TR (Table 1). The template used for each reaction was 0.1 ng semi-nested PCR product. Each 20 µl reaction consisted of 0.1 ng template, 1 x Buffer, 2.8 mM MgCl_2_, 200 µM each dNTP, 0.25 pM of the AS forward primer, 2.5 pM common reverse primer, 0.2 µl Amplifluor SNP primer FAM and 0.5U Thermostart DNA Polymerase. AS-PCR was performed using the Rotorgene-3000 real-time PCR thermocycler and consisted of an initial 8 cycles to allow for determination of background fluorescence at 95°C for 2 min, 56°C for 15 sec and 72°C for 10 sec, followed by 55 cycles of denaturation at 95°C for 20 sec, primer annealing at 56°C for 15 sec and extension at 72°C for 10 sec where the fluorescence was recorded. Samples were run against known standards, which were prepared by a number of reactions using different proportions of donor and recipient purified PCR products (100∶0; 50∶50; 95∶5; 99∶1; 99.9∶0.1; and 99.99∶0.01%). 100% porcine mtDNA was also run as a control for the AS forward primer to determine whether there was any non-specific primer binding and the degree of specificity and sensitivity of the AS-PCR reaction.

### Whole genome amplification and microsatellite analysis

Reconstructed embryos were subjected to whole genome amplification using the Genomiphi V2 DNA Amplification Kit (GE Healthcare, Buckinghamshire, UK), according to the manufacturers′ instructions. Firstly, an embryo in 1 µl water was added to 9 µl of sample buffer. The template DNA was denaturated at 95°C for 3 min then cooled to 4°C on ice. The reaction was performed at 37°C for 1.5 hr with 9 µL reaction buffer and 1 µl enzyme mix. The Φ29 DNA polymerase enzyme was inactivated by heating the sample at 65°C for 10 min then kept at -20°C for further analysis. Seven pairs of microsatellite makers (D1Mit206, D4Mit178, D8Mit242, D12Mit270, D16Mit146, D19Mit10 and DXMit170) on 7 different chromosomes were selected to analyse the reconstructed embryos. PCR amplification of these loci was performed in a 15 µL final volume containing 0.25 IU HotStart Taq DNA polymerase (Qiagen, Crawley, UK) in 1XPCR buffer, 0.2 mM dNTPs, 2.5 mM MgCl_2_, 0.2 µM each primer and 2ng of the whole genomic DNA amplification product. The amplification protocol specified initial denaturation for 15 min at 94°C, 40 cycles of 30 sec each at 94°C, 30 sec at the specific annealing temperature, as listed in Table 1, 30 sec at 72°C, followed by a final extension step of 72°C for 5 min. PCR products were subjected to electrophoresis on a 3% agarose gel at 80V for 2 h and the products were visualized under UV.

### Single embryo gene expression analysis

First strand complementary DNA was synthesized by priming with random hexamers. Single embryos were suspended in 10 µl solution consisting of 0.2 µg random hexamers, 0.02 µM DTT, 1 Unit RNase inhibitor. The hexamers were annealed by incubating the sample at 70°C for 5 min and then immediately quenched on ice. Reverse transcription was performed by the addition of 10 µl solution consisting of 1 µl 10 mM dNTPs, 1X buffer and 1 µl BioScript and incubated at 25°C for 10 min followed by another 40 min incubation at 42°C. 3 µl of product was used as template for target gene amplification. The PCR reactions consisted of 0.5 µM of each primer (see Table 1), 200 uM dNTPs and 2 mM MgCl_2_ and 1IU DNA polymerase (Roche, Lewes, UK) at 94°C for 4 min followed by 40 cycles of 94°C 20 sec, 30 sec at the specific annealing temperature (see Table 1), 30 sec at 72°C, followed by a final extension step of 72°C for 5 min.

### Microarray analysis

Total RNA was extracted from mESCs and MEFs using the RNAqueous 4PCR Kit (Ambion, Austin, TX, USA), according to the manufacturer′s protocol. Contaminating genomic DNA (gDNA) was eliminated by adding 5 µ l of 5x gDNA Elimination Buffer (SABiosciences, Frederick, MD, USA) and incubating at 42°C for 10 min. cDNA was synthesised using the RT^2^ First Strand Kit (SABiosciences, Frederick, MD, USA), according to the manufacturer′s protocol. Each 20 µ l reaction contained 1 µg RNA, 5x RT Buffer, Primer and External Control Mix, RT Enzyme Mix (SABiosciences) and sterile ddH_2_O. The reaction was performed at 42°C for 15 min and stopped by incubating at 95°C for 5 min. Prior to microarray examination, 91 µl of sterile dH_2_O was added to the cDNA mixture. mESC and MEF samples were analysed using the Mouse Embryonic Stem Cell RT^2^ Profiler PCR Array (SABiosciences). Each array profiles the expression of 84 genes associated with the maintenance of pluripotency and self-renewal using real-time PCR. Real-time reactions were performed in 96-well optical reaction plates (SABiosciences) by adding RT^2^ SYBR Green/ROX (SABiosciences) and sterile ddH_2_O to each reaction that had a final volume of 25 µ l. Real-time reactions were conducted on a 7500 Fast Real-Time PCR System (Carlsbad, CA, USA) and consisted of an initial denaturation step at 95°C for 5 min followed by 40 cycles of denaturation at 95°C and annealing/extension at 60°C for 1 min. Gene expression data were generated in the form of cycle threshold (Ct) values. Relative gene expression was calculated by the ΔΔCt method and normalized against the average Ct values of 5 housekeeping genes (Gusb, Hprt1, Hsp90ab1, Gapdh and Actb).

## Supporting Information

Table S1 Genes upregulated in mouse ESC extract. Only those genes with greater than a four-fold increase, when compared to MEF extract (control) have been included as this represents a significant change in expression.
(0.03 MB DOC)

Figure S1 NPC and histone H2A.X analysis in iSCNT embryos. (A) NPC assembly, indicated by arrows, in a porcine-porcine cloned blastocyst (i); and a murine-porcine cloned blastocyst (ii). NPC and DAPI are shown in green and blue, respectively. (B) Phosphorylation of histone H2A.X at serine 193 in a murine in vivo blastocyst (i); murine-porcine cloned blastocyst (ii); and porcine-porcine cloned blastocyst (iii). H2A.X and DAPI are shown in green and blue, respectively. Scale bar = 20 µm.
(0.72 MB TIF)
