# Supplementary material for: Interspecies Somatic Cell Nuclear Transfer Is Dependent on Compatible Mitochondrial DNA and Reprogramming Factors
Source: PLoS One. 2011 Apr 27;6(4):e14805. doi: 10.1371/journal.pone.0014805 (PMC3083390; doi:10.1371/journal.pone.0014805)
Supplement: Table S1 — Genes upregulated in mouse ESC extract. Only those genes with greater than a four-fold increase, when compared to MEF extract (control) have been included as this represents a significant change in expression. (0.03 MB DOC) [file pone.0014805.s001.doc]

**Supplementary Data**

Table SI: Genes upregulated in mouse ESC extract. Only those genes with greater than a four-fold increase, when compared to MEF extract (control) have been included as this represents a significant change in expression.

| **Gene** | **Fold Change** |
| --- | --- |
| Nr6a1 | 4.72 |
| Sox2 | 5.49 |
| Lefty2 | 6.35 |
| Il6st | 8.45 |
| Lifr | 10.61 |
| Dnmt3b | 13.38 |
| Gata6 | 16.57 |
| Nodal | 17.3 |
| Ednrb | 29.85 |
| Cd9 | 49.88 |
| Lefty1 | 55.18 |
| Grb7 | 91.23 |
| Nog | 109.43 |
| Zfp42 | 265.98 |
| Pou5f1 | 861.56 |
| Podxl | 2251.29 |
| Lin28 | 3719.78 |
| Kit | 130425.52 |
